# An analysis of the efficacy of universal PCR and BACTEC 9120 BD for identifying bacteremia in pediatrics

**DOI:** 10.3205/dgkh000509

**Published:** 2024-10-23

**Authors:** Azam Safarkhani, Fatemeh Shirkavand, Nafiseh Abdollahi, Nazanin Ahari Oskooie, Leila Azimi, Mohammad Rahbar, Abdollah Karimi

**Affiliations:** 1 Pediatric Infections Research Center, Research Institute for Children’s Health, Shahid Beheshti University of Medical Sciences, Tehran, Iran; 2 Department of Biology, Science and Research Branch, Islamic Azad University, Tehran, Iran; 3 Department of Microbiology, Reference Health Laboratories Research Center, Ministry of Health and Medical Education, Tehran, Iran

**Keywords:** bacteremia, BACTEC, molecular methods, phenotypic methods

## Abstract

**Background:**

Bloodstream infections (BSI) are serious diseases in pediatrics and can increase the rate of morbidity and mortality. Blood culture is time consuming and can have false negative results in some case such as the intracellular or fastidious bacteria. This study aimed to evaluate the PCR against automated blood culture with BACTEC.

**Materials and methods:**

In this observational cross-sectional study the blood samples of hospitalized children in Mofid Children’s Hospital with bacteremia signs from February to May 2023 were enrolled. The causative bacteria in bacteremia were identified by phenotypic and PCR methods.

**Results:**

150 blood samples were enrolled to identify the presence of bacteremia by BACTEC and PCR. 60% and 40% of samples have negative and positive results in both methods, respectively. PCR showed 100% sensitivity and specificity in detecting bacteremia compared to BACTEC. A variety of bacteria were identified by phenotypic and molecular methods and coagulase negative *Staphylococcus* (CONS) is the most of them.

**Conclusion:**

The rapid and accurate detection of bacterial pathogens with the high sensitivity and specificity compared gold standard method are the most important profits of molecular assay.

## Zusammenfassung

**Hintergrund:**

Blutstrominfektionen (BSI) gehört zu den schwerwiegenden Erkrankungen in der Pädiatrie und können die Morbidität und Mortalität erhöhen. Blutkulturen sind zeitaufwändig und können in manchen Fällen falsch-negative Ergebnisse liefern, wie z.B. bei intrazellulären oder anspruchsvollen Bakterien. Ziel dieser Studie war es, die PCR im Vergleich zur automatisierten Blutkultur mit BACTEC zu bewerten.

**Materialien und Methode:**

In der Querschnittsstudie wurden Blutproben von hospitalisierten Kindern im Mofid-Kinderkrankenhaus mit Anzeichen einer Bakteriämie von Februar bis Mai 2023 erfasst. Die Bakterien wurden mittels phänotypischer und PCR-Methode identifiziert.

**Ergebnisse:**

Es wurden 150 Blutproben untersucht. 60% bzw. 40% der Proben wiesen bei beiden Methoden negative bzw. positive Ergebnisse auf. Die PCR zeigte eine 100%ige Sensitivität und Spezifität beim Nachweis einer Bakteriämie im Vergleich zu BACTEC. Eine Vielzahl von Bakterien wurde durch phänotypische und molekulare Methoden identifiziert, wobei Koagulase-negative *Staphylococcus* (CoNS) den größten Anteil ausmachten.

**Schlussfolgerung:**

Die wichtigsten Vorteile des molekularen Tests sind der rasche und genaue Nachweis von bakteriellen Krankheitserregern mit hoher Sensitivität und Spezifität.

## Introduction

Nosocomial infections (NI) are related with different toxins or infectious agents such as bacteria, viruses or parasites that cause infection among patients admitted to the hospital [1]. The most common form of NI is blood infection or bacteremia [2] that increases in-hospital mortality and increases treatment costs, especially in ill newborns who are admitted to the NICU or ill children that in PICU [3], [4]. Patients with hemato-oncologic diseases are at high risk for developing bacteremia because they are often severely immunocompromised due to underlying disease, antineoplastic therapy, and/or hematopoietic stem cell transplantation. This infection associated with some kind of invasive device entering the venous blood system such as central vascular catheters (CVC) and lead to the central line-associated bloodstream infection [5], [6]. Bacteremia is now associated with significant morbidity and mortality in pediatrics and the appropriate and prompt treatment of this infection is necessary and has been shown to significantly reduce mortality [7]. In order to accurately identify the microorganisms responsible for these infections, blood culture stands out as a crucial diagnostic procedure. It is recommended that blood samples be collected just before initiating any empirical antimicrobial therapy. Nonetheless, the effectiveness of blood culture may be compromised in cases where patients have recently been on antibiotics or when dealing with slow-growing or intracellular micro-organisms, leading to delays and lower sensitivity in detection. Identifying microorganisms, particularly in blood samples, through the utilization of pathogen-specific or broad-spectrum PCR tests shows great potential. Furthermore, the advancements in Real-Time PCR technology come with a multitude of benefits when compared to conventional PCR methods. These advantages encompass rapid results, ease of use, accurate quantification abilities, and reduced chances of contamination [8]. The bloodstream infection (BSI) is an important infection in children, especially in hospitalized patients in PICU and can increase the rate of morbidity and mortality [9], [10]. BSI specially affects the outcome of children undergoing cardiac surgery, increasing complications and mortality [9]. For this reason, it is important to improve BSI diagnosis. So, the purpose of this research is to analyze results of PCR against of BACTEC in samples sent to the Children’s Infectious Research Center (PIRC) Laboratory at Mofid Children's Hospital.

## Materials and methods

### Sample preparation and set up

The research was conducted as an observational cross-sectional study at Mofid Children’s Hospital from February to May 2023. The study included collecting blood samples from hospitalized patients in the Pediatric Intensive Care Unit (PICU), Neonatal Intensive Care Unit (NICU), as well as patients in the transplant and hematology units showing symptoms of bacteremia like Fever and chills. The study was approved by the ethics committees of the research institute for children health, Shahid Behehshti University of Medical Science by approved ID: IR.SBMU.RICH.1402.009. 

### BACTEC process

BACTEC bottles (BD BACTEC™ 9120) were sent from wards prefilled with 5 ml of whole blood to laboratory of PIRC. The bottles were incubated immediately upon receipt in the microbiology laboratory in accordance with the manufacturer’s recommendation. Bottles flagged as positive by the BACTEC system were sub-cultured in MacConkey and blood agar Following that identified by gram stain and other biochemical tests like oxidase, catalase, TSI, and DNase.

### Molecular detection

300 µl of each BACTEC samples were used for extracting total DNA by commercial extraction kit (SIMBIOLAB). The extracted samples were used for conventional PCR for detecting 16S rRNA in all samples for confirmation of present of bacteria in samples. In the next step for detecting the *Staphylococcus aureus, Enterococcus spp., Escherichia coli, Klebsiella pneumoniae* and *Enterobacter cloacae* conventional PCR as well as Real Time PCR was used for detecting *Neisseria meningitidis, Hemophilus influenzae* and *Streptococcus pneumoniae*. The primers were shown on Table 1 (Tab. 1). 

### Statistical analysis

The data was analyzed using statistical software SPSS version 27. P value of less than 0.05 was accepted at the level of significance.

## Results

150 blood samples from hospitalized children under 18 years old were enrolled to identify the presence of bacteremia by both BACTEC and PCR methods. 90 (60%) out of 150 samples had negative results in both BACTEC and PCR assay as well as 60 (40%) samples were positive. This result indicates a strong correlation between results of PCR and BACTEC, with PCR demonstrating 100% sensitivity and specificity in detecting bacteremia compared to bacteria culture as a gold standard technique.

The bacteria were identified by phenotypic identification following a positive BACTEC. Molecular analysis was then conducted on samples that tested positive for 16srRNA in PCR. The most identified bacteria in both methods was coagulase negative *Staphylococcus* (CONS) (Table 2 (Tab. 2)). 

The phenotypic approach was unable to identify any of co-infection, but PCR analysis confirmed the presence of co-infections with *K. pneumonia* and *E. cloacae* 2 cases.

Two co-infections with *Klebsiella pneumoniae* and *Enterobacter cloacae* were detected by PCR. While the *Citrobacter* spp. and *Klebsiella pneumoniae* were identified in phenotypic methods in these two samples. 

In one instance, *Staphylococcus aureus* was identified using a phenotypics methods but PCR detected the CONS. 

A study reported the presence of *Pseudomonas* spp. and *Acinetobacter* spp. using a phenotypic method, but the genus of the species was not clearly defined. However, through PCR analysis, it was possible to identify *P. aeruginosa* and *A. baumannii*. Specifically, 11 samples were identified as *P. aeruginosa*, 4 as non-*aeruginosa Pseudomonas*, 8 as *A. baumannii*, and 3 as non-*baumannii Acinetobacter* using PCR analysis.

## Discussion

Due to the low sensitivity of blood cultures, it is crucial to develop new approaches to quickly identify the bacteria responsible for infections, especially in children [9]. Traditional techniques like phenotypic tests can be replaced with innovative methods such as multiple PCR because the phenotypic methods are time-consuming and occasionally have false negative results [9]. Molecular technologies enable quick and precise identification of the pathogen responsible for infectious diseases in a much shorter duration compared to conventional methods [11], [12].

The findings of this study have validated the superior accuracy of PCR as a molecular diagnostic tool for detecting bacteremia. PCR can amplify various regions of the bacterial 16S ribosomal ribonucleic acid (rRNA) and accurately identify the causative bacteria. Our study demonstrated that PCR conducted directly on whole blood samples correlates well with BACTEC, consistent with findings from previous studies [13], [14]. A study has highlighted a crucial need for the ability to access PCR test results for antimicrobial stewardship purposes within hospital settings [15], [16]. The findings from these studies [15], [16] align with our research results. 

The latest findings from this study reveal that there was no discernible distinction in detecting Gram-positive and Gram-negative bacteria responsible for causing bacteremia. All samples exhibited a strong correlation with the outcomes obtained from the BACTEC bacterial culture technique.

PCR is more effective at identifying bacteria responsible for bacteremia than BACTEC. Sometimes, determining the genus of bacteria requires complex testing procedures and the use of resources therefore, the laboratory can identify specific strains of bacteria by PCR. For instance, the laboratory mentioned in this research study can only identify *Pseudomonas* spp. or *Acinetobacter* spp. However, by using PCR, we can easily detect the genus of bacteria. Another crucial aspect is the use of molecular assays for accurately identifying bacteria in co-infections. Our findings indicate that PCR successfully identified two co-infections in bloodstream infections, phenotypic identification methods did not. The key advantages of using molecular testing for bacteremia diagnosis include rapid and accurate detection of causative bacteria, making PCR a viable alternative to bacterial culture and phenotypic identification methods.

## Conclusion

As technology continues to advance, molecular techniques are playing a more prominent role in standard microbiological testing and have brought about a significant change in how we deal with bloodstream infections. Being able to quickly diagnose severe infections is crucial for starting the right treatment promptly and avoiding the unnecessary use of antibiotics, which can lead to adverse effects and increased medical expenses. The key benefits of utilizing molecular methods for identifying bacterial pathogens and antimicrobial resistance genes include faster and more cost-effective results.

## Notes

### Authors’ ORCID

- Azam Safarkhani: 0009-0003-3325-498X
- Fatemeh Shirkavand: 0009-0001-5207-4883
- Nafiseh Abdollahi: 0000-0002-1820-4680
- Nazanin Ahari Oskooie: 0009-0008-5647-7525
- Leila Azimi: 0000-0002-7216-2530
- Mohammad Rahbar: 0000-0002-3070-3108
- Abdollah Karimi: 0000-0002-4225-0097

### Ethical approval

The ethical approval number of this study is (IR.SBMU.RICH.REC.1402.009) from the Ethics Committee of the Research Institute for Children’s Health, Shahid Beheshti University of Medical Sciences, Tehran, Iran. 

### Data Availability Statement

The data presented in this study are available upon reasonable request from the corresponding author.

### Funding

Research reported in this publication was supported by Researcher Grant Committee under grant number [43006041] from the Shahid Behehsti University of Medical Sciences, Tehran, Iran.

### Competing interests

The authors declare that they have no competing interests.

## Figures and Tables

**Table 1:**
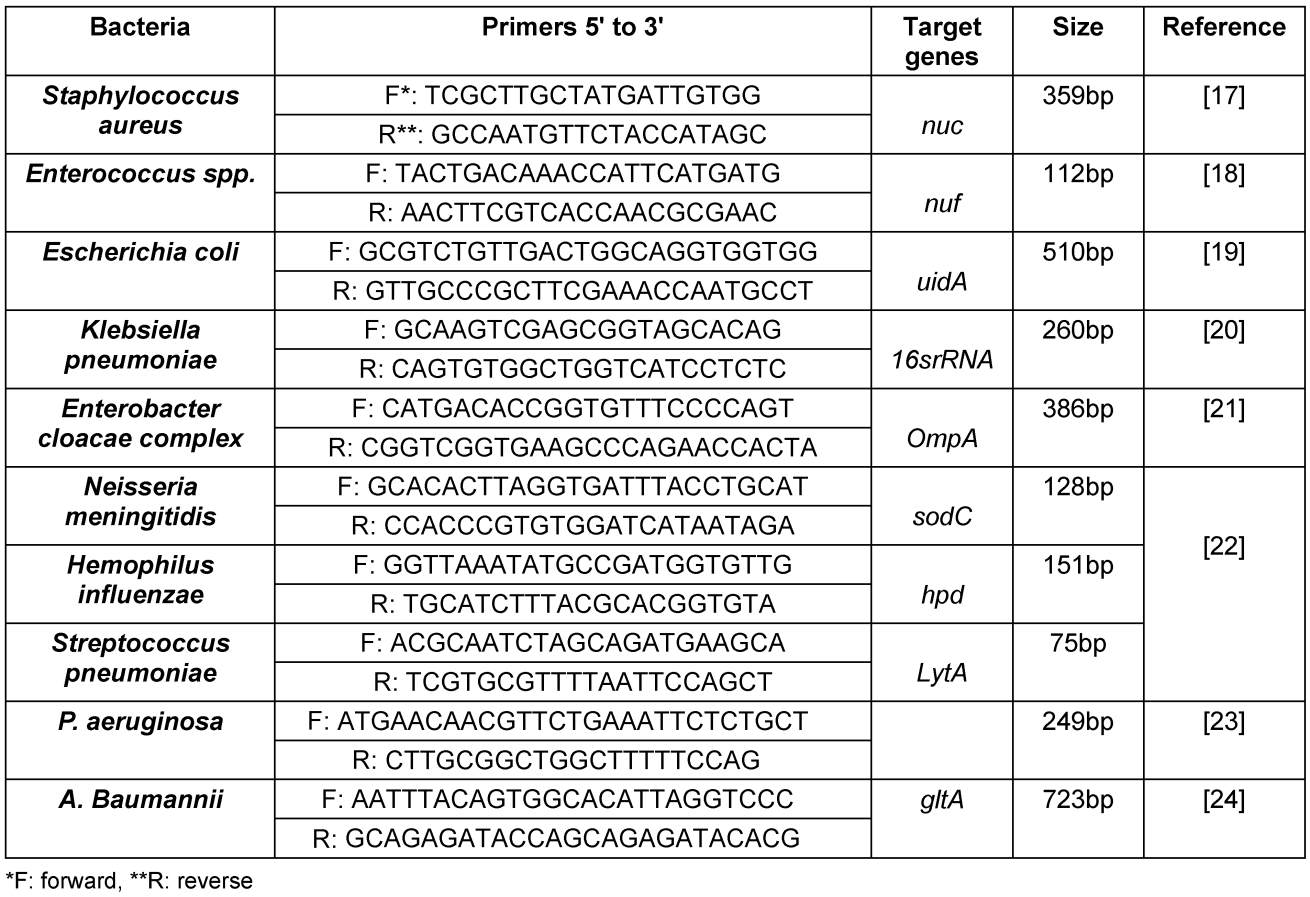
Primers used in this study

**Table 2:**
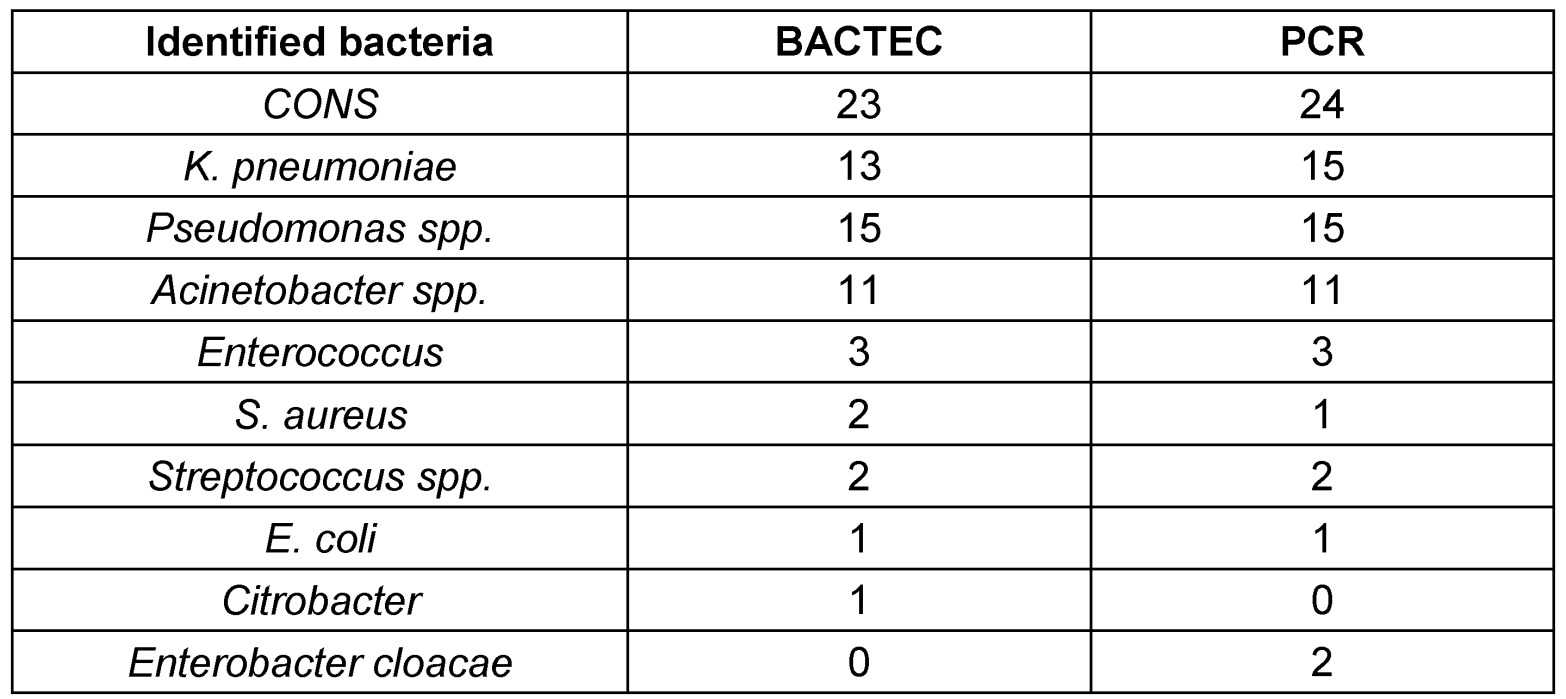
Identified bacteria after positive BACTEC and PCR
